# Patient coordination during the COVID-19 pandemic in the Amsterdam region: effects on capacity utilization and patient flow

**DOI:** 10.1186/s12913-025-12311-w

**Published:** 2025-02-17

**Authors:** E. Berkeveld, M. D. F. Rhebergen, F. W. Bloemers, H. R. Zandbergen, G. G. van Merode

**Affiliations:** 1 Amsterdam University Medical Center Location Vrije Universiteit Amsterdam, Department of Trauma Surgery, De Boelelaan 1117, 1081 HV Amsterdam, the Netherlands; 2 Network for Acute Care Noord-Holland Flevoland, Meibergdreef 9, Amsterdam, the Netherlands; 3 Amsterdam University Medical Center Location University of Amsterdam, Department of Trauma Surgery, Meibergdreef 9, Amsterdam, the Netherlands; 4 Amsterdam University Medical Center Location University of Amsterdam, Department of Cardiothoracic Surgery, Meibergdreef 9, Amsterdam, the Netherlands; 5 Care and Public Health Research Institute, Maastricht University, Maastricht, 6200 MD The Netherlands; 6 Maastricht University Medical Centre+, P. Debyelaan 25, 6229 HX Maastricht, The Netherlands

**Keywords:** Decision making, Surge demand, Capacity pooling, Crisis management, Health care policy

## Abstract

**Background:**

To manage COVID-19 surge demand, Dutch regional and national task forces were installed to coordinate a proportionate patient distribution. This study examined the effect of centralized COVID-19 patient coordination on hospital capacity utilization during the pandemic.

**Methods:**

A retrospective observational double cohort study compared intra- and interregional patient coordination by the regional task force ROAZ Noord-Holland Flevoland. Coordination was compared to a simulated scenario without coordination based on a queueing model during two time periods from January 1, 2021, until May 1, 2021 and from August 1, 2021, until December 1, 2021. Daily data on patient ICU and clinical COVID-19 patient transfers, number of admissions, and capacity were assessed. The primary outcome was hospital capacity utilization.

**Results:**

Overall, 1,213 patients were transferred both within the eleven regional hospitals and outside the region during cohort I and 528 patients during cohort II. During the first cohort, eight hospitals (ICU patients) and two hospitals (clinical patients) showed a utilization factor exceeding 100% without coordination which reduced to below 100% with coordination. During the second cohort, utilization factors exceeding 100% varied between the scenarios with and without coordination. In both cohorts, the majority of hospitals that showed a utilization factor below 100% in the scenario without coordination, showed an increased utilization factor in the scenario with coordination.

**Conclusion:**

This retrospective double cohort analysis based on regional coordination of COVID-19 patients and a simulated scenario of absent regional coordination, identified that load-balancing of COVID-19 care demand generally resulted in an improved distribution of utilization among hospitals. In a crisis, we suggest a swift upscale from local, regional to national centralized coordination activity to enable inter and intra-regional patient coordination at an early stage. Future research is recommended to explore the applicability of coordination for other patient categories to benefit from regional centralization during a crises.

## Background

High quality and accessible health care is essential. During the COVID-19 pandemic, worldwide healthcare systems were strained rapidly, with patients requiring immediate medical attention and resources for an extended period [1]. Due to the crisis character of the increased care demand, novel approaches were created and implemented to manage the call on surge capacity successfully. In the Netherlands, to prevent hospitals and regions from being overwhelmed, task forces were installed to coordinate the COVID-19 patient distribution on regional and national level [2–4].

In region Noord-Holland Flevoland, a task force was promptly installed based on an existing managerial network for acute trauma care (Regionaal Overleg Acute Zorg (ROAZ)) [5]. Local crisis coordinators were appointed on hospital level, adhering to close lines of communication with the task force. This way, a collaborative framework was established in which patient coordination could be organized [3]. Given the fluctuating COVID-19 care demand, flexibility was critical for the task force and local hospital crisis coordinators to react promptly to the changing circumstances. Through this almost real-time coordination and in-hospital flow management, uncertainty in COVID-19 demand as a collective was minimized [6].

A proportionate distribution of COVID-19 patients among hospitals and regions was aimed throughout the regional collaborative framework. Therefore, the COVID-19 patient coordination process was guided according to a ´fair share´ principle to provide equal load-balancing of the COVID-19 care demand. Based on a hospital´s size, catchment area and specific expertise function, hospitals were required to provide care for a certain share of the total admitted patients [3]. The number of admitted patients was obtained twice daily [2]. Following results would indicate which hospitals exceeded or receded their required fair share and the task force would use these results as a directive in their coordination. For example, if hospital A had 25 admitted COVID-19 patients while according to the fair share model they should provide care for 30, the task force would in theory aim to coordinate five COVID-19 patients to hospital A. Likewise, if hospital B had 20 COVID-19 patients admitted while their fair share would indicate 15, the task force would aim to relocate five patients to hospitals who were below the fair share such as hospital A. In case of fair share disparities between regions, the regional and national task force would coordinate possible inter-regional patient transfers [3].

In addition, the task force would obtain information from local hospital crisis coordinators concerning the current capacity status. Situations that affected the capacity were taken into consideration to create full situational awareness. This way, the fair share results based upon static number of admitted patients twice a day, were enhanced to almost real time information. As a result, the task force could make informed coordination decisions [3]. Situations affecting capacity included a high number of patients suspected of COVID-19 at the Emergency Department (ED) with pending test results, a strained ED with COVID-19 patients whose necessity for admission is still to be medically determined, a hospital declared temporary arrival stop of COVID-19 patients in ED, a temporary closure of a COVID-19 clinical ward by the hospital (e.g., due to an infectious disease outbreak such as MRSA) or pressing staffing capacity issues.

Currently, the attention to pandemic preparedness is extensive, with possibilities being explored by the World Health Organization (WHO) for an international treaty supporting pandemic preparedness, prevention, and response [7]. In the Netherlands, the Ministry of Health, Welfare, and Sport supports attention to means to respond decisively and adequately to possible future infectious outbreaks [8]. In addition, in large-scale incidents, disasters, or crises, inter- and intraregional patient transfer coordination should be easily reactivated [9]. This study aimed to assess the effect of centralized decision-making by our regional task force during the COVID-19 crisis. Therefore, the effects of centralized COVID-19 patient coordination on capacity utilization and patient flow during the pandemic were examined.

## Methods

### Study design

A retrospective observational double cohort study was conducted based on the coordination process involved in intra and inter-regional patient transfers coordinated by the regional ROAZ task force Noord-Holland Flevoland. The effect of COVID-19 patient coordination was compared to a simulated scenario in which no coordination would have occurred. The scenario wherein patient coordination occurred was characterized by centralized decision-making on regional level and on hospital level. In contrast, the simulated scenario in which no coordination occurred, was characterized by centralized decision-making solely on hospital level.

Cohort I included all data from January 1, 2021, until May 1, 2021. Cohort II included data from August 1, 2021, until December 1, 2021. Daily data on patient transfers, number of admissions, and capacity were quantitatively assessed for clinical and ICU COVID-19 patient transfers. Data was retrieved from the daily-shared admission and capacity correspondence between the hospital crisis coordinators and the task force. Data were subsequently analyzed anonymously as per the type of hospital. The primary outcome consisted of the utilization of capacity.

### Method of simulating queues and bed occupancy

#### Queuing and pooling

A *Queueing system* is defined as servers that offer services to customers. Unavailability or occupancy of servers results in customers waiting in one or more queues [10]. To optimize a queueing system in terms of effectiveness, it is aimed to increase a server's utilization and decrease service variability. A method to achieve this consists of pooling. Pooling indicates the replacement of multiple elements by one single element. Pooling can be carried out in all three aspects of the queueing system, that is, “pooling queues (the demand), pooling tasks (the process) or pooling servers (the resources)” [11]. Through pooling, arrival patterns, service times and utilization can be influenced. Moreover, the need for coordination and capacity in a queueing system can be affected. More specifically, an increase in pooling would result in a higher necessity for coordination in the particular queueing system in which the pooling takes place, whereas it decreases the necessity for coordination with other queueing systems the pooled system is connected to [12].

#### Assumptions in queueing systems

The time between customer arrivals is described by the *inter-arrival time*, which consists of $\frac{1}{\lambda }$ per unit of time in which $\lambda$ is the *arrival rate* per unit of time. *Service processes* are expressed by the *service times* of servers, the type of servers and the number of servers. Queues can be shared between servers or a queue can belong to only one server. Service times are expressed by the mean service time $\frac{1}{\mu }$ per unit of time. $\mu$ stands for the *service rate* at a particular server [12].

In a queueing system, a relation is assumed between the *service rate* $\mu$ and the *capacity* that is available. A rate of customer arrival less than the rate of service, $\lambda <\mu$, is conditional for a controlled queueing system. In case of a queueing system with one server, efficiency is determined by the difference between the rate of customer arrival ($\lambda$) and the rate of service ($\mu$). Therefore, the degree of utilization of a server is determined by $\frac{\lambda }{\mu }$. Likewise, underutilization, measured as $\frac{\mu -\lambda }{\mu }$, can indicate a system's inefficiency and can be caused by fluctuation in the demand, straining the queueing system. This underutilization can be relieved by coordination activities [12].

Two forms of demand can be distinguished; either *uncertain* or *stochastic.* Uncertain demand is characterized by demand caused by an unknown process. The simulated scenario without coordination can be characterized by uncertain demand, as the hospitals do not influence how much demand they will receive. In contrast, demand is characterized as stochastic when it occurs random and the random process is identified [13]. The real scenario with coordination can therefore be characterized by stochastic demand, in which coordination functions as the random identified process. Therefore, times of arrival between customers are assumed to be stochastic and independent from one another. Also, the customer service times are assumed to be stochastic and independent of one another [10].

### Data analysis

The patient coordination process was considered as pooling of the demand and capacity. As the *capacity* per hospital slightly variated, the mean available capacity was calculated and used. For *capacity* the *utilization factor* was determined. To calculate the utilization factor, the total occupied bed hours were divided by the total available capacity hours per hospital. If higher than one, the queuing process is considered instable. This could lead to overutilization of the real capacity. The number of times this situation occurs is used to calculate the chance that the hospital is completely occupied. To determine the simulated effects in the scenario of no patient coordination a pooling in queuing network approach was used [11].

In all scenario’s a M/M/c queueing model was used where *c* is the number of beds with arrival rate λ and service rate μ. Exponential distributions were used for arrival and service times. The inter-arrival times and service times were derived on the basis of Little law [14] $L=\lambda W$

With

*L* = average number of items in the queuing system,

*W* = average waiting time in the system for an item, and

λ = average number of items arriving per unit time

Mathematica [15, 16] was used to code and analyze the queueing models. Inter-arrival times and service times were calculated and used in units of hours.

## Results

### Translation into queueing models

**Fig. 1:**
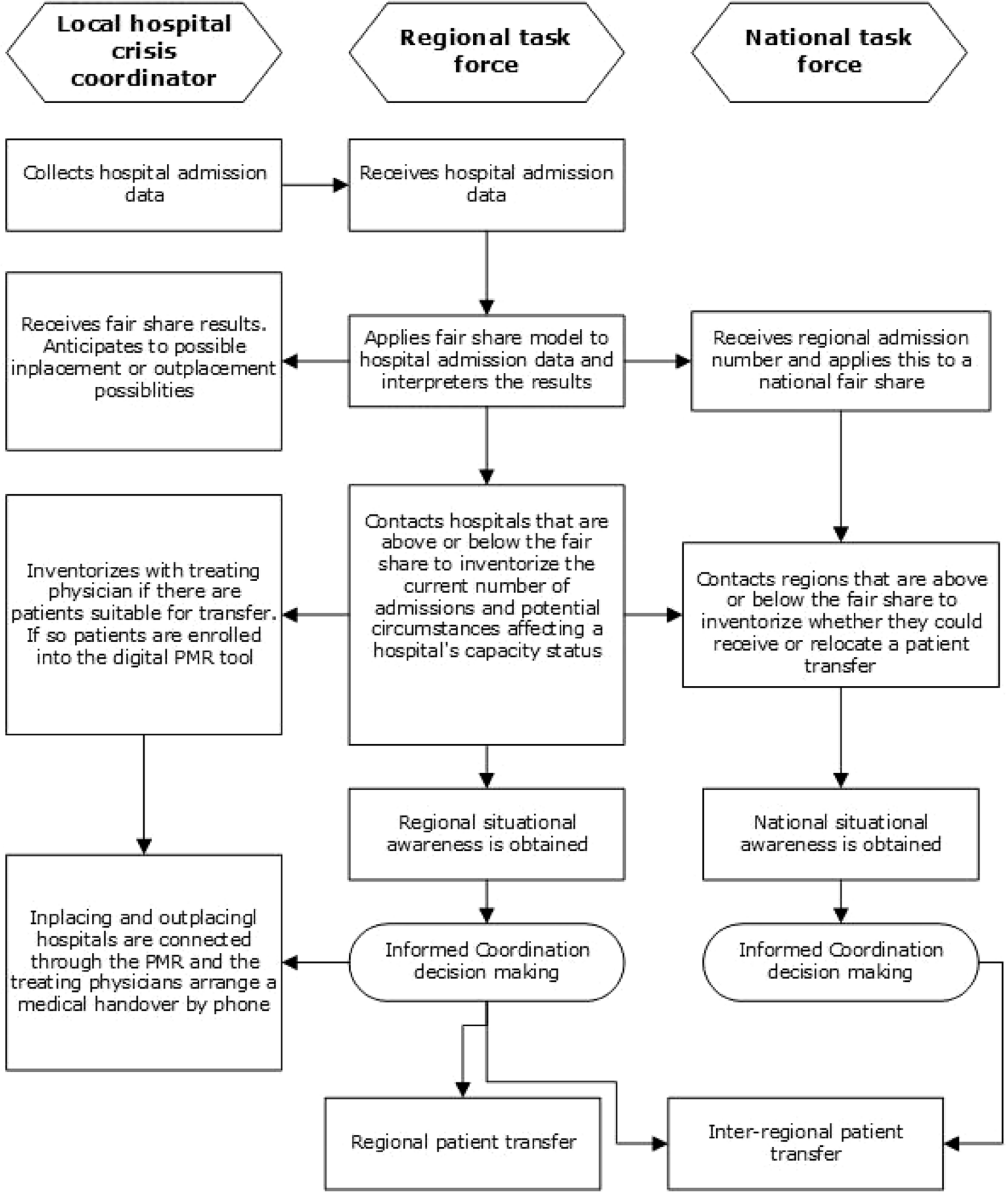
Operational coordination process flowchart. Rectangles represent tasks and arrows represents the introduction of a next task after the previous one is completed. Abbreviations: PMR, patient movement request

The framework in which the COVID-19 patient distribution was organized collectively shared the COVID-19 demand and the consequent capacity. The total sum of admitted COVID-19 patients was aimed to be distributed proportionately among all hospitals (Fig. 1).

**Fig. 2:**
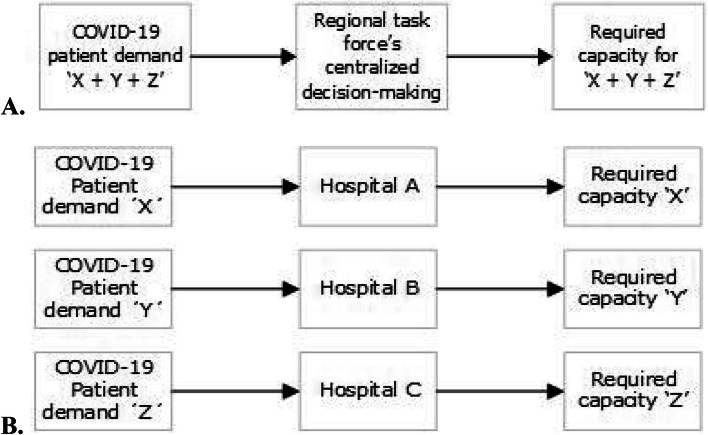
**A** Depiction of the demand, process and resource flow with coordination. **B** Depiction of demand, process and resource flow without coordination

In terms of service systems, the COVID-19 patient coordination can therefore be seen as a queueing model which both the queue (i.e., the COVID-19 demand) and the servers (i.e., the required COVID-19 capacity) pooled. Figure 2A shows this queueing model in which the COVID-19 demand and corresponding capacity are pooled. In comparison, in a regular situation without patient distribution, the demand is not pooled and each hospital requires to provide capacity for the demand that is brought upon them (Fig. 2B).

### Quantification

During the first cohort, a total of 1,213 patients were transferred. The majority of transfers, 694 patients, were relocated outside the region (57.2%), whereas 495 patients were transferred within the region (40.8%), and 24 patients were received from outside the region (2.0%). In contrast, during the second cohort, 528 patients were transferred. The majority of transfers, 317 patients, occurred within the region (60.0%), while 110 patients were transferred to another region (20.8%), and 101 patients were received from another region (19.2%).

The mean hospital capacity for ICU and clinical COVID-19 patients was higher during the first cohort than the second cohort (Table 1). Both for ICU patients and clinical patients, the number of admissions were higher during cohort I compared to cohort II. During cohort I, except for the first (academic) hospital, all hospitals had more frequent admissions of ICU and clinical patients in the (simulated) scenario than in the (real) scenario with coordination. During the second cohort, the ICU admissions were more frequent in the scenario without coordination for six hospitals. Regarding the admissions of clinical patients, two hospitals showed a higher frequency in the scenario without coordination during the second cohort.

**Table 1:** Mean COVID-19 admission capacity

	**ICU Capacity**		**Clinical Capacity**	
	Cohort I	Cohort II	Cohort I	Cohort II
**Hospital**				
**1**	31.4	14.4	64.0	16.0
**2**	16.3	5.8	32.4	13.9
**3**	12.8	4.4	33.2	14.8
**4**	8.4	2.6	18.1	8.1
**5**	10.2	3.3	24.5	10.5
**6**	5.4	2.6	11.2	5.4
**7**	11.7	3.5	38.4	16.6
**8**	6.4	1.7	20.3	11.6
**9**	4.1	1.3	15.1	6.1
**10**	3.0	2.3	9.1	4.9
**11**	1.6	0.0	11.7	5.1

*ICU* Intensive care unit

**Fig. 3:**
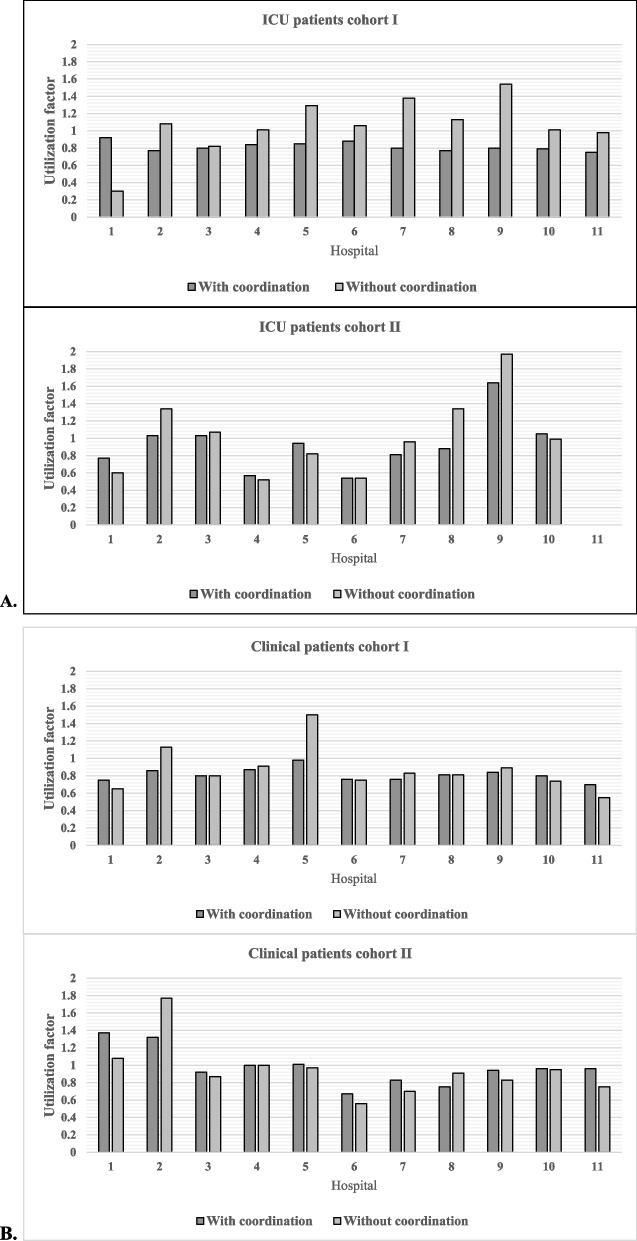
**A** COVID-19 ICU patients during cohort 1 (above) and cohort 2 (below). ‘With coordination’ are real figures. ‘Without coordination’ simulated figures based on the real demand. **B** COVID-19 clinical patients during cohort 1 (above) and cohort 2 (below). ‘With coordination’ are real figures. ‘Without coordination’ simulated figures based on the real demand

During cohort I, eight out of eleven hospitals had a utilization factor above one in the scenario without coordination of ICU patients (Fig. 3A). In the scenario with coordination of ICU patients, the utilization factors of these hospitals decreased below one (ranging from 0.77 to 0.88). Specifically, four out of the five largest hospitals in the region (numbers 2, 5, 7, and, 8) showed utilization rates reducing to below one in case of the coordination, while they had a utilization rate above one when no coordination occurred. Three hospitals (number 1 academic, number 3 large, number 11 small) had a utilization rate remaining below one in both scenario´s. In the academic hospital (number 1), an increase in utilization factor from 0.30 till 0.92 was observed.

During cohort II, four hospitals had a utilization factor above one without coordination of ICU patients that reduced in the scenario with coordination. Hospital number eight had a reduction of utilization factor below one, whereas hospitals two, three and nine had a reduced utilization factor that remained above one. Hospital number ten showed an increased utilization rate above one in case of coordination (from 0.99 to 1.05).

Regarding the clinical patients during cohort I, two hospitals (numbers 2 and 5) showed a utilization factor above one in the scenario without coordination (Fig. 3B). In the scenario with coordination, the utilization factor of these hospitals changed to below one. The other nine hospitals had a utilization rate below one without coordination which remained below one in case of coordination.

During cohort II, three hospitals showed a utilization factor above one for clinical patients in case of no coordination. In the scenario with coordination, hospital two showed a reduced utilization rate (from 1.77 to 1.32), hospital number four showed an unchanged utilization rate (from 1.00 to 1.00), and the academic hospital (number 1) showed an increased utilization rate (from 1.08 to 1.37). Hospital number five showed an increased utilization rate from below one in case of no coordination to above one in case of coordination (from 0.97 to 1.01). Except for hospital number 8, all remaining hospitals showed an increased utilization rate which remained below one in case of coordination compared to no coordination.

## Discussion

This study assessed the effect of COVID-19 patient coordination by a regional task force on capacity utilization and patient flow. Overall, distributing COVID-19 patients among hospitals and regions through coordination aimed to minimize strain on COVID-19 and regular healthcare capacity. During the first cohort, coordination resulted in the prevention of COVID-19 patient overcrowding on a regional wide level for COVID-19 ICU patients and to a lesser degree for clinical COVID-19 patients. During this time, all hospitals whose capacity utilization exceeded 100% in the scenario without coordination showed a reduction of the utilization rate to below 100% in the scenario with coordination. Moreover, in both cohorts, coordination resulted in an optimized capacity utilization for the majority of hospitals that showed a low utilization rate without coordination. This effect is demonstrative of the reduced uncertainty of demand for the individual hospital caused by coordination [12]. These results show the effect of more equal distribution of patients through load-balancing and demonstrate the beneficial effect that capacity pooling can have during a crisis.

Several factors presumably contributed to the effectiveness of COVID-19 patient coordination during the crisis. First, the distributed patients consisted of a homogeneous patient population (i.e., COVID-19 disease as the primary admission reason). All patients were medically examined and diagnosed before they were eligible for coordination. Through this, uniform and unambiguous patient categories could be distributed to any hospital's COVID-19 bed within and outside the region. To respond to the need for surge capacity, ICU and clinical cohort departments were installed as a temporary surge capacity for the COVID-19 patient group. This way, clarity of hospital admission insight was achieved, aiding the coordination process' efficiency through an overall clear provision of information. Second, the adhered fair share provided a directive for the proportionate distribution. Due to this model supporting coordination, applied both on regional and national levels, hospitals and regions were collectively prevented from being overwhelmed. Third, the task force performing the coordination was led by physicians experienced in managing Mass Casualty Incidents. Their technical competence and the task force's unbiased position resulted in expert power, as comes with an integrating role defined by Lawrence and Lorsch [17]. Both on local and regional levels, (almost) real-time decision-making occurred by healthcare professionals. We believe healthcare professionals are eminently capable to fulfill decision-making roles during a crisis, mirroring the instinctive-led decision-making perspective of healthcare professionals as described by Weick [18]. They are experienced in application of a detailed approach and in managing crises situations in their regular work.

Besides, various external factors developed over the course of the Dutch epidemic that could have influenced the observed differences between both cohorts. As a response to the COVID-19 infection, the Ministry of Health, Welfare and Sport installed a vaccination campaign against the COVID-19 infection starting January 8th 2021 [19]. While vaccination practices started during the first cohort, they were completed at the start of the second cohort. Additionally, a shift in the predominant COVID-19 variant occurred over the summer of 2021 as the Delta variant replaced the Alpha variant, causing an increased transmissible, though less severe variant to be predominant during the second cohort [20, 21]. These differences might explain the fewer transfers and required capacity for COVID-19 patients observed in cohort 2. Moreover, due to a high number of COVID-19-positive infections prior to the first cohort, a high crisis activity within the entire regional collaborative framework and on a national level was in full swing. In contrast, the second cohort commenced after a period of relatively low positive infections, in which crisis activity state was low and national transfers were not possible up till the first half of the second cohort. In line with this, fewer patients were transferred during cohort 2 compared to cohort 1. To manage this demand during a crisis effectively, an early upscale of coordination from (sub-)local, regional to national level appears critical. Thereby increasing the ability to rapidly act upon an increased surge demand.

In the fair share system, the bed utilization was used as a proxy for resource utilization, but when a particular hospital had not reached the fair share level, it could incidentally be excused for admitting an additional patient. A hospital’s realtime COVID-19 capacity could be influenced by a variety of factors (e.g., staffing shortage or a strained ED) and was determined by a crisis activity state reflecting a proportionate minimum capacity. The crisis activity state was evaluated three times weekly in national discussions [3], and supported by demand forecasting of national trends of COVID-19 positive infections [22]. Buffer times to meet the required increase in capacity of 48–72 h were adhered. At the same time, coordination process continued on a 24/7 base. This discrepancy might explain why for some hospitals the occupancy exceeded the capacity, as observed in the results of cohort 2. Moreover, the academic hospital (number 1) specifically functioned as a regional last resort to which often transfers had to be sent to if smaller sized hospitals, who are inherently more prone to strain due to their limited surge capacity, exceeded their fair share. Likewise, results from cohort 2 show that improved utilization was mainly observed in larger sized hospitals. The larger size allows hospitals to have more capacity besides the fair share which is preventive for over utilization.

Furthermore, to effectively manage the inherent demand uncertainty during a crisis, the centralized task force requires an optimally designed framework and effective processes. Generally, the way an organization is designed is pivotal for the performance effectiveness. The coordination process as described in Fig. 1 and followed by the taskforce entailed a continuous balance between the current demand, the subsequently derived fair share results and a hospital’s capacity. Large amounts of highly detailed information from within the regional differentiated framework required daily processing to adequately conduct the real time decision making. According to Galbraith’s theory on organizational design, three strategies to minimize information processing include increasing the possibility to preplan, increasing the flexibility to accommodate for the lack of preplan, or decreasing the level of quality of the performed task [22].

The framework of COVID-19 coordination mirrors Galbraith´s strategies by either increasing the capacity for information processing or limiting the requirement to process information [23]. The fair share was based on the number of admitted patients, gathered twice daily, and enhanced through the current capacity status provided by local hospital crisis coordinators. This direct response ensured the collective management of the current COVID-19 demand (i.e., the admitted number of COVID-19 patients). The procedure of data collection by all hospitals resembles Galbraith’s integrating mechanism ‘coordination by rules or programs’ as it concerns a routine and predictable task [23]. The following real time decision making is consistent with Galbraith’s second integrating mechanism ‘coordination by targets or goals as it resembles a planning approach to reach certain outcomes [23]. The overall sum of regional admissions was shared with the national task force. This information-processing approach constitutes an hierarchical structure from local to regional and national level, which forms the third integrating mechanism [23]. Moreover, effectiveness of complex organizations further depends to which degree tasks are outlined. According to Lawrence and Lorsch task differentiation refers to the degree of which tasks are divided into subsystems. Task integration refers to the unity of effort in the subsystems to achieve the organizational task [17]. Task structure can be divided according to several integrating and differentiating factors to reach the collective aim for load-balancing (Table 2).

**Table 2:** Integrating and differentiating factors

Integrating factors	Differentiating factors
Sharing the hospital admission data twice daily with the region to create collective awareness	Autonomous COVID-19 patient care delivery
Applying the fair share model to the hospital admission data to obtain proportionate results for each hospital	Collection of hospital admission data twice daily of COVID-19 patients admitted in the clinical department or ICU
Interpretation of the fair share results by the task force so that it functions as a coordination directive	Interpretation of the fair share results by the local hospital crisis coordinators from which they could derive the expectation concerning relocating patient transfers, receiving patient transfers, or no action required
Enrollment of a request for patient transfer by the local crisis coordinator in the digital PMR tool that is received by the task force	Performing a medical handover by the treating and receiving physician as the final step before the transfer process
Contact by telephone between the task force and local hospital crisis coordinators from hospitals whose admissions exceeded or receded their fair share. This contact aimed to obtain insight into the current number of admissions, the degree of strain affecting the hospital's continuity of care, and possible circumstances affecting the hospital's capacity, all contributing to informed coordination decision-making by the task force	Arranging transport by Emergency Medical Services from the relocating to the receiving hospital
Coordination decision-making by the task force	

´Integrating factors´ refer to unity of effort in the subsystems to achieve the organizational task. ´Differentiating factors´ refer to the degree of which tasks are divided into subsystems. *PMR* Patient Movement Request

Regional coordination inherently entails a form of centralization on regional and hospital level. Inherently, due to involved capacity decisions regarding the installed COVID-19 clinical and ICU cohort departments, centralization at the department level was also involved. Despite the hospital centralization inherently involved in regional coordination, we have no insight into to what extend this hospital level centralization actually occurred. Besides, in the simulated scenario without patient coordination, only centralization on hospital level occurred. As both scenarios include centralization on hospital level, the results of our analysis do not compare regional centralization with decentralization.

### Limitations

This study analyzed the effect of coordination over the entire duration of each cohort. Therefore, although the coordination processes consisted of a realtime changing environment, the results do not indicate a change of utilization factor on a daily level but solely on the entire cohort’s duration. Also, manual registration of capacity was used by local crisis coordinators, which inherently has its risk for subjective errors in data registration. In terms of generalizability, the concept of patient distribution among hospitals through coordination might require adjustments to accommodate the geographical challenges in systems with vast distances. Furthermore, ideally, it would be preferable to incorporate individual patients´ data on admission and discharge dates, including transfer data, and also for patients outside of the fair share. However, we accommodated our study design to the data available. This way, our model provides insight into the effects of capacity pooling -through central coordination- on occupancy rates. Pre-COVID data was not available for comparison as a counterfactual. However, such comparison would be challenged by differences in patient type, decision-making and data collection. Additionally, while only known in retrospect, external circumstances between the first and second cohorts affect the model’s predictability and reliability. The actual decision-making that would have occurred in a real scenario without coordination remains unknown. Moreover, our model used stochastic inter-arrival times and service times. The assumption that if a patient would be ready for discharge, the patient’s bed would become immediately available for a new patient was applied. However, in real life, several other scenarios might occur. First, if the bed is freed by discharging a patient, this discharge should often be planned. In this way, a new patient can be assigned to this bed before the discharge has actually taken place. It is often unclear to the health professional when the patient can be discharged. This phenomenon is called positional entropy. This concept has been developed by Gong et al. to measure the uncertainty of the actual position of a product or part during manufacturing [23]. Winasti et al. have applied this concept to measure the uncertainty of the actual state of recovery or treatment in a hospital in relation to the discharge time [24]. Positional uncertainty can also be used to conceal that the patient is ready for discharge but the department’s staff or management do not want a new patient. Therefore, a patient is occupying a bed that could be used for a new patient. Second, discharge and admission decisions might not be taken synchronously. For example, discharge could occur continuously at an ICU, but admission decisions twice a day in a regional coordination model. Third, one may speculate about that this ‘gaming’ can take place within and between hospitals and influences the admission possibilities and utilization.

## Conclusion

This retrospective double cohort analysis based on regional coordination of COVID-19 patients and a simulated scenario of absent regional coordination, identified that load-balancing of COVID-19 care demand generally resulted in an improved distribution of utilization among hospitals. In a crisis, we suggest a swift upscale from local, regional to national centralized coordination activity to enable inter and intra-regional patient coordination at an early stage. Future research is recommended to explore the applicability of coordination for other patient categories to benefit from regional centralization during a crises.

## Data Availability

Original data remain available and access may be provided upon reasonable request. Contact: e.berkeveld@amsterdamumc.nl.

## Abbreviations

ROAZ: Regionaal Overleg Acute Zorg
ED: Emergency Department
WHO: World Health Organization
